# Cost savings of anti-TNF therapy using a test-based strategy versus an empirical dose escalation in Crohn's disease patients who lose response to infliximab

**DOI:** 10.3402/jmahp.v3.29229

**Published:** 2015-10-30

**Authors:** Xavier Roblin, Alain Attar, Michel Lamure, Bernard Savarieau, Pierre Brunel, Gérard Duru, Laurent Peyrin-Biroulet

**Affiliations:** 1 Department of Gastroenterology, University Hospital of Saint Etienne, Saint-Etienne, France; 2 Department of Gastroenterology, IBD, Nutritional Support, Hôpital Beaujon, Clichy, France; 3 University Claude Bernard, Lyon 1, Lyon, France; 4 Nukleus, Paris, France; 5 Pharmacy Department, University Hospital of Saint Etienne, Saint-Etienne, France; 6 Inserm U954 and Department of Gastroenterology, Université de Lorraine, Nancy, France

**Keywords:** infliximab, pharmacokinetics, adalimumab, Crohn's disease, cost saving

## Abstract

**Background:**

The use of pharmacokinetics is associated with cost savings in anti-tumor necrosis factor (anti-TNF) therapy, but the long-term cost savings in a large cohort of Crohn's disease (CD) patients are unknown.

**Aim:**

The goal of this study was to compare the cost of anti-TNF therapy in two cohorts of CD patients losing response to infliximab, one using a test-based strategy and one an empirical dose escalation.

**Methods:**

We used a selected mathematical model to describe the trajectories of CD patients based on a discrete event system. This design allowed us to track over a given period a double cohort of patients who moved randomly and asynchronously from one state to another, while keeping all the information on their entire trajectory. Both cohorts were modeled using state diagram parameters where transition probabilities from one state to another are derived from literature data. Costs were estimated based on the French health care system.

**Results:**

Cost savings among the 10,000 CD patients using a test-based strategy were €131,300,293 at 5 years. At 5 years the mean cost saving was €13,130 per patient. The direct cost of the test had no impact on the results until the cost per test reached €2,000.

**Conclusions:**

A test-based strategy leads to major cost savings related to anti-TNF therapy in CD.

The monoclonal antibodies against tumor necrosis factor (TNF) – infliximab (IFX) and adalimumab (ADA) – are increasingly used to treat inflammatory bowel disease (IBD) that is refractory to standard medication (1). In the COIN study, health care costs were mainly driven by medication costs, most importantly by anti-TNFα therapy, while hospitalization and surgery accounted only for a minor part of the health care costs (2).

A significant proportion of primary responders to anti-TNF therapy will lose response over time or may become intolerant to these agents (3, 4). Despite the advent of new biologics such as vedolizumab, therapeutics is still limited for IBD patients. Accordingly, international guidelines recommend optimizing anti-TNF therapy by shortening the interval and/or increasing the dose before switching to another biological agent. Such empirical dose escalation may not be optimal in terms of cost.

A growing body of evidence indicates that therapeutic drug monitoring may be used to optimize disease outcomes (5–7).

## Methods

Changes in a patient's state challenge physicians to provide the most suitable treatment during the course of IBD. A systems thinking approach is needed when modeling the paths of patients treated for IBD (8). Different events may occur along a patient path. They can be numerous and can occur at random from one patient to another. The modeler is then faced with an adaptive complex system in which there are so many possible combinations that methods able to handle this complexity are needed.

For IBD, this complexity makes it difficult to use a decision tree, which would be unreadable and unmanageable. Moreover, methods based on differential equations are unusable because the nature of the problem is such that no equation, however sophisticated, describes its behavior.

Using a model-based Markov chain is also not possible for two main reasons. First, temporal state changes vary from one patient to another. Second, the entire path of the patient must be kept in mind, and this is incompatible with Markov chain modeling.

For IBD, we need to use a model based on a discrete event simulation described and computationally modeled by means of the life sequence charts (LSCs), which are an extension of statecharts. In a conceptual framework, the modeler sees the patient as a reactive object whose behavior is characterized by its response to events dispatched from outside its own context and is also affected by its past. Statechart formalism was introduced by Harel's team in 1985. Statecharts have since evolved into LSCs. After being used in manufacturing problems, their use and the use of discrete event simulation appeared in the life sciences in the early 2000s (9), with increasing success.

Here, we compared two large cohorts of CD patients who had lost response to anti-TNF therapy and were being managed using either a test-based strategy to determine simultaneous anti-IFX antibody and residual IFX levels or an empirical dose escalation up to 5 years of follow-up using the LSC method. The aim of our study was to compare the cost of anti-TNF therapy in these two cohorts.

**Fig. 1:**
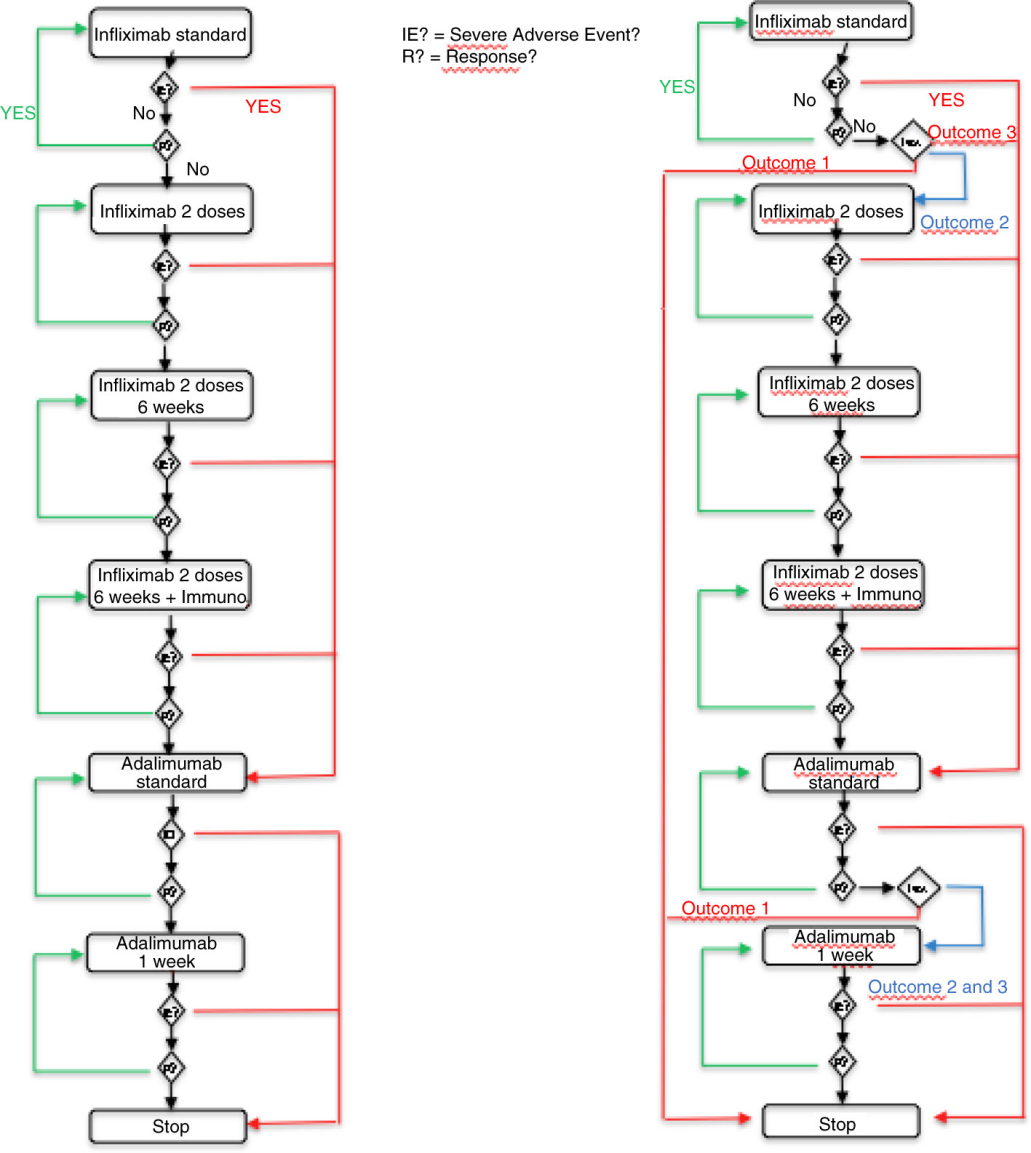
Using Harel's statechart diagrams, description of changes in patients’ states over time according to events that occurred and treatment changes made.

The simulation model involved creating two virtual cohorts of patients in a discrete events system. The first one corresponded to the current protocol for the treatment of CD. The second one modeled a virtual cohort of patients following a modified protocol wherein a diagnostic test is included. By using Harel's statechart diagrams, we described how any patient's state can change over time according to events that occur and treatment changes that are made. Figure 1 shows two diagrams corresponding to these statecharts. The left part of the diagram corresponds to the patient's path in the current protocol. The right part of the diagram corresponds to the protocol in which a diagnostic test is introduced. Based on these two diagrams, a software tool was developed to simulate the dynamics of each of the two cohorts. Different simulation time horizons were considered: 1 year, 3 years, and 5 years. According to medical practice, there are several time units: 8 weeks and 6 weeks for treatment using IFX; 2 weeks and 1 week for treatment using ADA. Two cohort sizes – 3,000 and 10,000 – were simulated for the three time horizons.

The diagnostic test is assumed to have three possible outcomes:

1. High concentration of biologic>3 µg/mL and negative anti-drug antibody (ADAb) (Outcome 1)
2. Low concentration of biologic<3 µg/mL and positive ADAb (Outcome 2)
3. Low concentration of biologic<3 µg/mL and negative ADAb (Outcome 3)

Our simulation model provided, for each patient in the cohort, the entire history of his or her path, including events that occurred in response to treatment, treatment-related adverse events, and all changes in treatment for the whole simulation period. Based on the output of the simulation tool, the number and duration of the courses of treatment (a course of treatment is the use of a drug at a given dose for a specific duration) were computed, patient by patient. The costs entailed by these courses of treatment in the two cohorts could then be computed.

### Data entered into the model

The model parameters, namely the different probabilities of events that may occur, were determined according to the literature data. Table 1 shows the list of events considered and the associated probabilities of occurrence. These probabilities were derived from annual probabilities found in the literature and recalculated to take into account the different time units used in this work: the difference in the lengths of the courses of treatment between the two cohorts.

**Table 1:** Model inputs according to clinical events

	Probability		
		
Probability of one event at each consultation	Minimum	Maximum	References
Loss of response to IFX therapy at maintenance dose without any adverse event	0.019	0.023	Gisbert (4)
Serious adverse event on IFX therapy	0.026	0.032	Hanauer (10) Cummings (11)
Loss of response after optimization of IFX therapy (double dose) without any serious adverse event	0.098	0.104	Katz (12)
Severe adverse event on IFX (10 mg/kg)	0.026	0.032	Hanauer (10) Cummings (11)
Loss of response after optimization of IFX therapy (double dose) every 6 weeks without any serious adverse event	0.075	0.079	Chapparo (13)
Severe adverse event on IFX (10 mg/kg) every 6 weeks	0.021	0.023	Hanauer (10) Cummings (11)
Loss of response after optimization of IFX therapy (double dose) every 6 weeks without any serious adverse event with addition of IS therapy	0.075	0.079	Chapparo (13), Vande Casteele (14) Leclerc (15)
Severe adverse event on IFX (10 mg/kg) every 6 weeks with addition of IS	0.019	0.024	Leclerc (15)
Loss of response to ADA therapy at maintenance dose without any adverse event	0.007697	0.008689	Billoud (3) Colombel (16) Baert (17) Sandborn (18, 19)
Serious adverse event on ADA therapy	0.006729	0.007603	Colombel (16)
Loss of response on ADA therapy every week at maintenance dose without any adverse event	0.003832	0.004354	Sandborn (20) Roblin (21)

IFX, infliximab; ADA, adalimumab; IS, immunosuppressive.

These parameters were used to simulate the occurrence of various events, patient by patient, throughout the observation period.

### Modeling and statistical analysis

The software tool used (Anylogic^®^) generated the entire path of all patients in the cohort for the period considered, based on events that occurred in the history of these patients. These paths were then analyzed using other software (R scripts), in order to calculate the following for each patient:

1. The number of courses of anti-TNF treatment a patient had during follow-up
2. The timing of these courses of treatment

This analysis provided complete information on the changes in each patient over time until the end of preoperative use of anti-TNF therapy.

Data from medical and economic databases (PMSI, a French database) were used to estimate the cost of treatment with anti-TNF for each patient in both cohorts, thus enabling comparisons. We estimated the cost of each type of treatment used (IFX 8 weeks, IFX 6 weeks, IFX 6 weeks with immunosuppressant, ADA 2 weeks, ADA 1 week) and calculated the total cost of treatment for each patient, depending on the number of courses of each type of treatment (Table 2). To carry out statistical tests, the last part of the work was to conduct a sensitivity analysis by randomly varying the probabilities (uniform distribution) of occurrence of various events according to specified intervals given by experts from the isolated estimates found in the literature.

**Table 2:** Treatment costs through social insurance

8 weeks infliximab	8 weeks infliximab double dose	6 weeks infliximab double dose	6 weeks infliximab double dose+immunosuppressive	2 weeks adalimumab	1 week adalimumab	Adalimumab induction
€1840.18	€3318.61	€3318.61	€3348.42	€306.72	€306.72	€1884.20

**Fig. 2:**
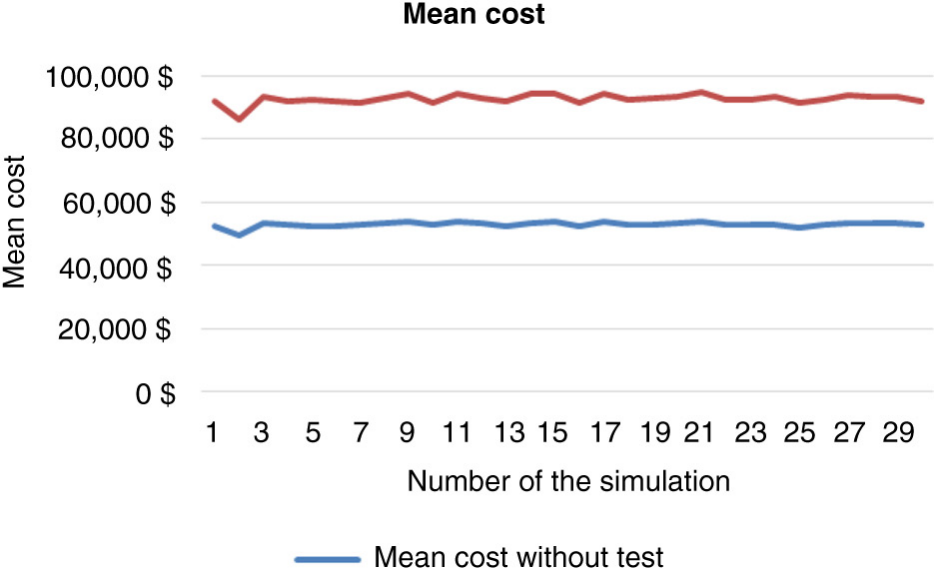
Mean anti-TNF-therapy cost distribution by simulation (10,000 patients, 5 years).

**Fig. 3:**
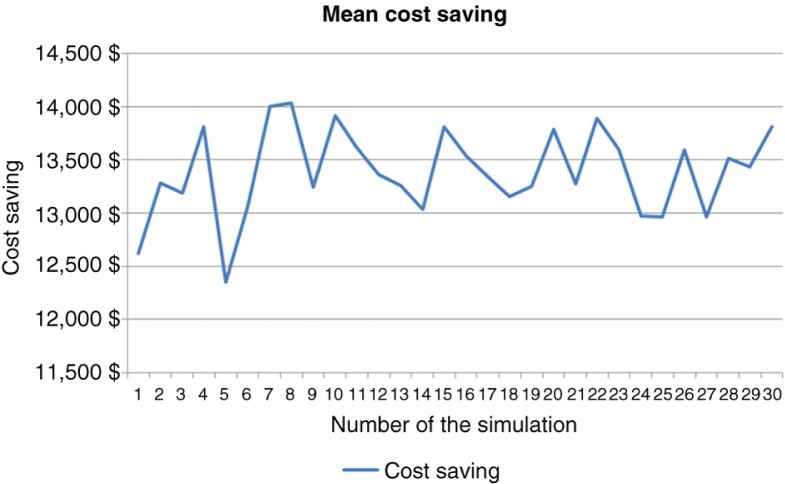
Difference in mean costs of anti-TNF drugs between clinical scenarios 1 and 2 (10,000 patients, 5 years).

A total of 3,000 replicates of 30 simulations indicated results-based costs, which, using bootstrap techniques, enabled determination of the mean costs. Standard deviation and confidence interval were computed in each cohort and a comparison test was performed. The results of these simulations are shown in Table 3 and Figs. 2 and 3.

**Table 3:** Sample of 30 simulations using the bootstrap technique (10,000 patients, 5 years)

Number of the simulation	Mean cost without test (€)	Mean cost with test (€)	Cost saving (€)	# tests
1	52,378	39,759	12,619	15,792
2	49,778	36,494	13,284	15,553
3	53,302	40,114	13,188	15,717
4	52,782	38,976	13,807	16,129
5	52,411	40,064	12,347	15,671
6	52,429	39,360	13,070	16,062
7	52,756	38,750	14,005	16,080
8	53,514	39,484	14,030	15,964
9	53,893	40,651	13,241	15,436
10	52,759	38,842	13,917	16,176
11	54,040	40,422	13,618	15,394
12	53,244	39,883	13,362	15,688
13	52,498	39,241	13,258	16,144
14	53,660	40,624	13,036	15,431
15	54 100	40,286	13,813	15,514
16	52,426	38,889	13,537	15,941
17	53,806	40,470	13,336	15,533
18	52,854	39,696	13,158	15,814
19	53,139	39,884	13,254	15,714
20	53,543	39,758	13,785	15,848
21	53,969	40,695	13,275	15,365
22	53,118	39,228	13,890	16,159
23	53,085	39,491	13,594	15,807
24	53,066	40,091	12,975	15,683
25	52,204	39,242	12,962	16,046
26	52,999	39,402	13,597	15,973
27	53,397	40,431	12,966	15,475
28	53,472	39,957	13,515	15,838
29	53,423	39,990	13,434	15,507
30	52,782	38,976	13,807	16,102

The probabilities of treatment failure (loss of response [LOR] or drug intolerance) among primary responders that were estimated following a comprehensive literature search are reported in Table 1.

### Clinical scenario in the empirical dose escalation group

In the empirical dose escalation group, the dose of IFX was first increased from 5 to 10 mg/kg every 8 weeks in CD patients who had lost clinical response to IFX. In case of clinical relapse, the interval between IFX infusions was shortened to 6 weeks. Then, in case of clinical relapse despite IFX dose increase, an immunosuppressant was initiated in combination with anti-TNF (22). Patients who lost response to combination therapy were switched to ADA therapy (160 mg, Week 1/80 mg, Week 2 induction regimen and 40 mg every other week in primary responders). Finally, in patients who lost response to ADA 40 mg every other week, anti-TNF treatment was optimized to 40 mg weekly. The probabilities of treatment success following empirical drug optimization were estimated following a comprehensive literature search (see Table 4).

**Table 4:** Probabilities of treatment success following empirical drug optimization at every consultation

Probabilities of clinical response after optimization	Minimum	Maximum	References
After optimization to IFX 10 mg/kg q8	0.896	0.902	Katz (12)
After optimization to IFX 10 mg/kg q6	0.921	0.925	Chapparo (13)
Addition of IS therapy	0.921	0.925	Chapparo (13), Vande Casteele (14), Leclerc (15)
Switch to ADA induction (160 mg, Week 1/80 mg, Week 2) followed by maintenance dose of 40 mg every other week	0.9913	0.9923	Billioud (3), Colombel (16), Baert (17), Sandborn (18, 19)
After optimization to ADA 40 mg weekly	0.9956	0.9961	Sandborn (20), Roblin (21)

IFX, infliximab; ADA, adalimumab; IS, immunosuppressive.

### Clinical scenario in the test-based strategy

Table 5 gives the probabilities of the three outcomes of the diagnostic test, decisions based on trough levels and antibodies to IFX, and the efficacy of every therapeutic decision based on literature data.

**Table 5:** Probabilities of the three outcomes of the diagnostic test, decision based on trough levels and antibodies to IFX, and efficacy of every therapeutic decision based on literature data

	Probability	Decision	Efficacy at 1 year	References
High TRI	0.50	Switch drug class	N/A	Paul (23), Sands (24), Yanai (25)
Low TRI without positive ATI	0.29	IFX optimization	80%	Paul (23), Afif (26), Vande Casteele (14)
Low TRI with detectable ATI	0.21	Switch to ADA	75%	Paul (23), Afif (26), Yanai (25)

IFX, infliximab; ADA, adalimumab; TRI, trough levels of IFX; ATI, antibody to IFX.

In the presence of antibody to IFX (ATI) without trough levels of IFX (TRI) (21%), it was decided to switch to another anti-TNF treatment. In the absence of both ATI and TRI (29%), therapeutic optimization with the same anti-TNF agent was initiated. For patients with high trough levels of anti-TNF regardless of ATI (50%), it was decided to change to a drug with a different mechanism of action or to consider surgery. Percentage response following such treatment decisions was estimated based on the studies by Paul et al. (23), Yanai et al. (26), and Afif et al. (25). In patients with LOR on IFX, with low TRI and positive ATI, the switch to ADA was associated with a clinical response in 75% of cases. In patients with low TRI without ATI, there was 80% clinical response after optimization of IFX.

For IBD patients presenting LOR on ADA maintenance treatment, an algorithm proposed by Roblin et al. formed the basis for a test-based strategy (21).

Table 6 gives the probabilities of the three outcomes of the diagnostic test, the decision based on ADA trough levels (TRA) and antibodies (AAA), and the efficacy of every scenario based on literature data (21).

**Table 6:** Probabilities of the three outcomes of the diagnostic test, decision based on trough levels and antibodies to ADA, and efficacy of every therapeutic decision based on literature data

	Probability	Decision	Efficacy at 6 months and 1 year	References
High trough levels of ADA	0.43	Switch drug class	N/A	Roblin (21)
Low TRA without positive AAA	0.24	ADA optimization	67 and 57%	Roblin (21) Baert (17)
Low TRA with detectable AAA	0.33	Switch to IFX	80 and 57%	Roblin (21)

IFX, infliximab; ADA, adalimumab; TRA, trough levels of ADA; AAA, antibodies to ADA.

In the presence of AAA without TRA (33%), it was decided to switch to another anti-TNF treatment. In the absence of both AAA and low or undetectable TRA (24%), therapeutic optimization with ADA therapy was implemented. In patients with high TRA regardless of AAA (43%), it is advisable to switch to a drug with a different mechanism of action. Percentage response following such a treatment decision was estimated based on the algorithm of Roblin and colleagues (21). In patients with LOR on ADA at maintenance dose with low TRA and positive AAA, a switch to IFX was associated with a clinical response in 80% of cases at 6 months and in 57% of cases at 1 year. In patients with low TRA without AAA, the clinical response after optimization of ADA was 67%.

### Cost of anti-TNF therapy in the model

Calculations were made using the costs in France according to our health care system. The treatment cost for IFX therapy was €492.81 for a 100 mg dose and the mean cost of one infusion of IFX was (GMH 28Z172) €361.75. For ADA therapy, the direct cost of treatment was €417.05 for 40 mg.

Trough levels of anti-TNF and concentrations of antibodies to anti-TNF were measured using the Lisa Tracker Premium Infliximab enzyme-linked immunosorbent assay (ELISA) kit (Theradiag, Marne la Vallée, France) at a cost of €100/unit (source manufacturer). This assay was developed to reduce low-affinity binding of immune complexes or interfering molecules such as the rheumatoid factor. The use of specific buffers for both the binding and washing steps allows very efficient capture of free molecules. Trough levels were considered undetectable for a concentration<0.1 µg/mL. The detection level reported by the manufacturer was>10 ng/mL. Antibodies to anti-TNF were defined as positive with this cutoff. Only one test was performed in our model for the first loss of IFX treatment response and a second test was performed in patients presenting LOR to ADA.

## Results

### Comparative cost savings of anti-TNF therapy in the two strategies in 10,000 patients

**Fig. 4:**
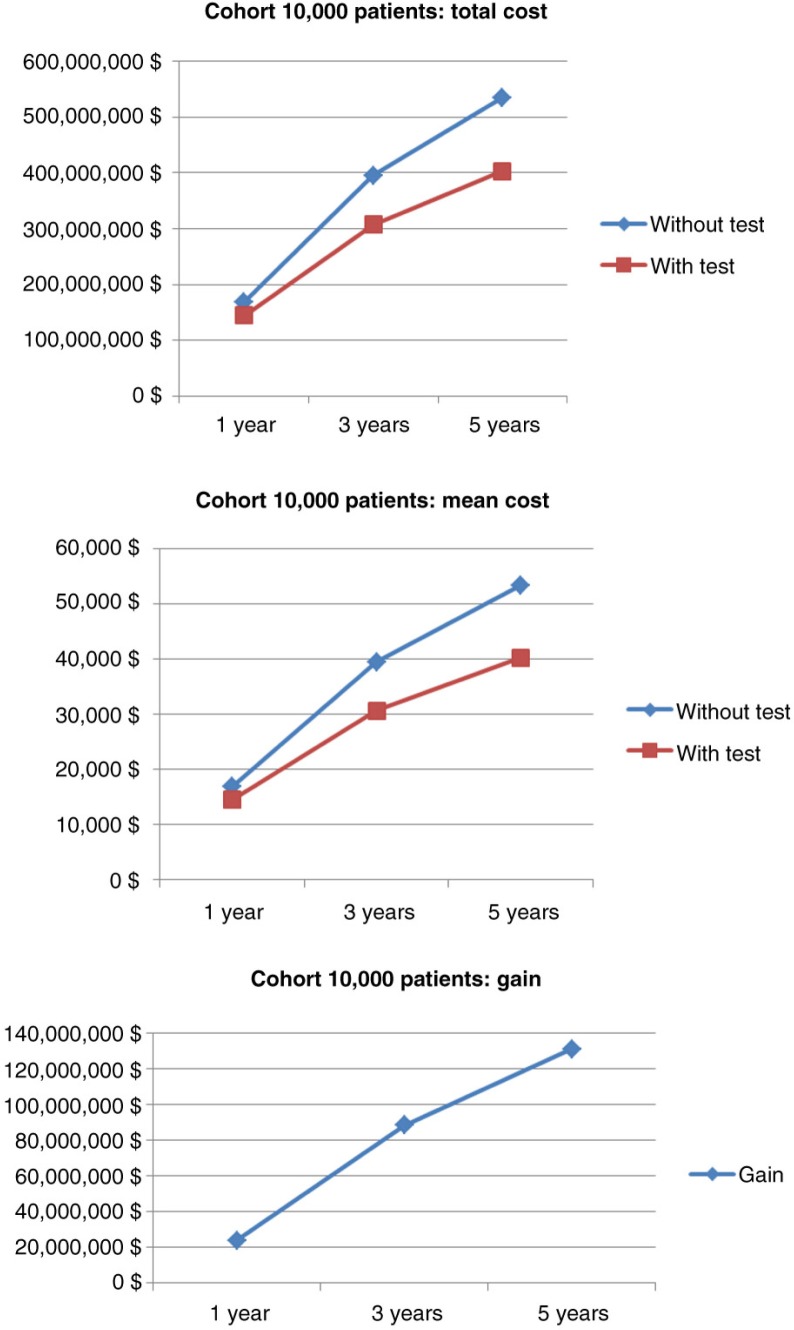
Comparative costs of anti-TNF therapy for the two strategies in 10,000 CD patients.

**Fig. 5:**
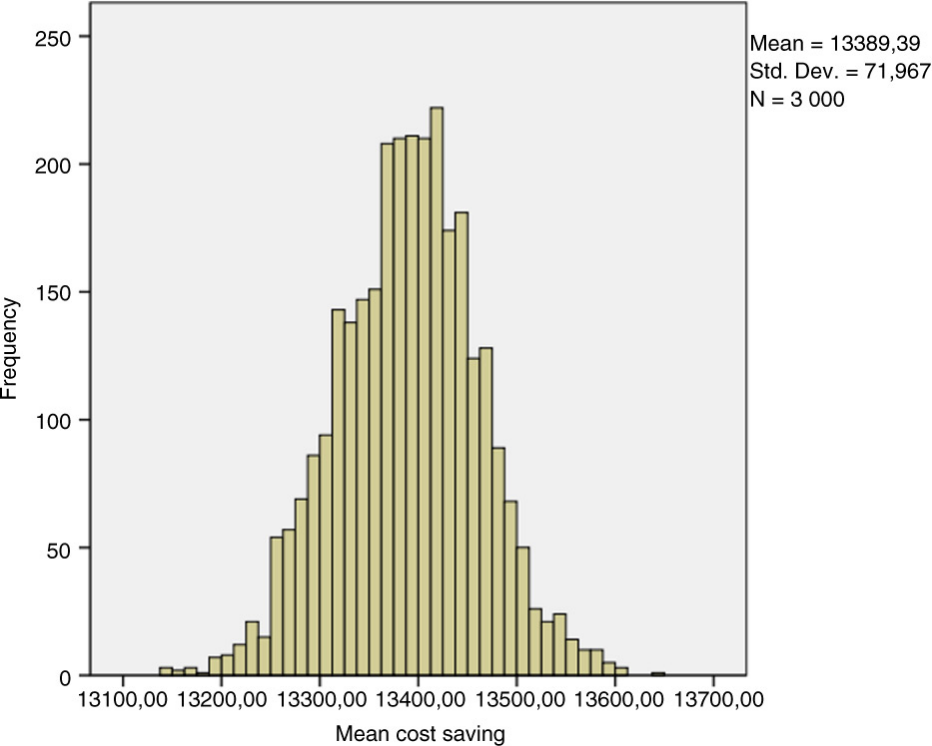
Bootstrapped histogram of cost savings (10,000 patients, 5 years).

Simulation for 10,000 patients indicated a respective overall cost of anti-TNF therapy at 1, 3, and 5 years of €168,488,150 (mean €16 849/patient; SD €2,797), €395,345,359 (mean €39,535/patient; SD €12,250), and €534,580,087 (mean €53,458/patient; SD €20,910) in the empirical dose escalation group (Fig. 4). In the test-based strategy group, these figures were respectively €144,640,532 (mean €14,464/patient; SD €4,240), €306,756,467 (mean €30,676/patient; SD €14,098), and €403,279,794 (mean €40,328/patient; SD €21,976) at 1, 3, and 5 years (Fig. 4). Hence, the cost savings of anti-TNF therapy were €23,847,619 (mean €2,385/patient; SD €5,080 – representing 14.1% of total costs), €88,588,892 (mean €8,859/patient; SD €18,677 – representing 22.4% of total costs), and €131,300,293 (mean €13,130/patient; SD €30,335 – representing 24.5% of total costs) at 1, 3, and 5 years, respectively (Figs. 4 and 5).

### Comparative costs of anti-TNF therapy in the two strategies in 3,000 patients

**Fig. 6:**
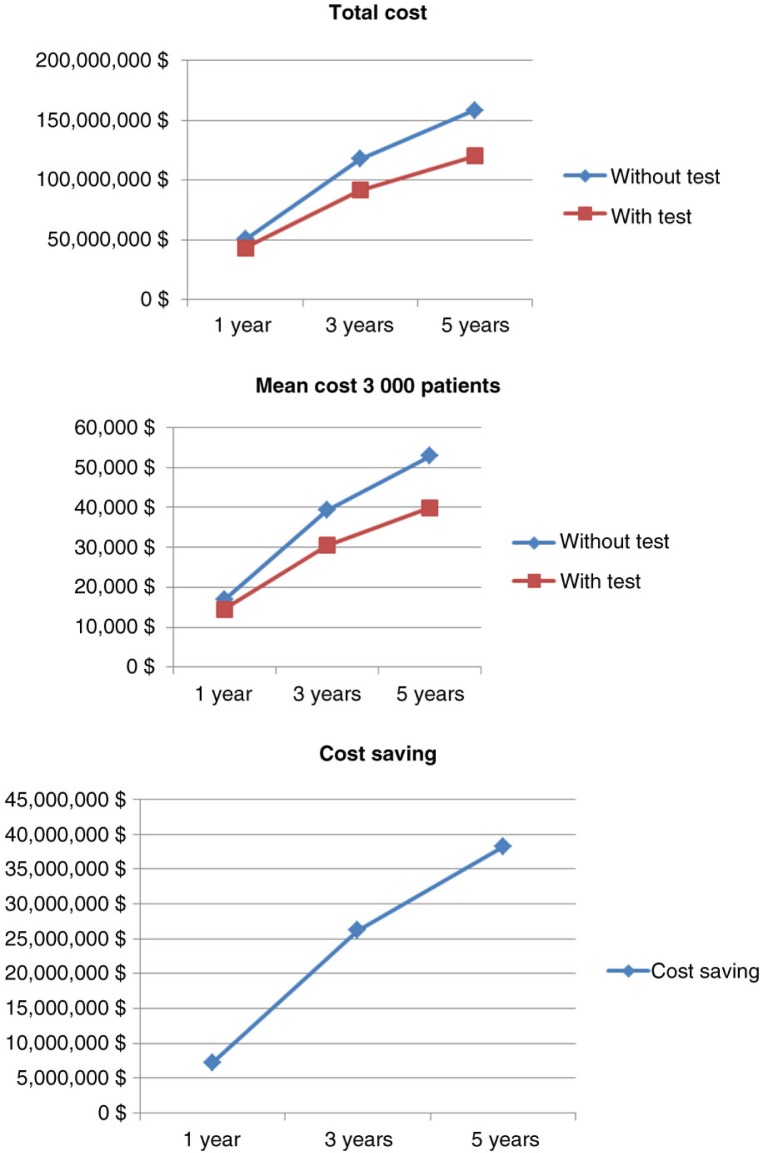
Comparative costs of anti-TNF drugs for the two strategies in 3,000 CD patients.

In the cohort of 3,000 CD patients, there were also dramatic cost savings among patients managed by the test-based strategy (Table 7 and Fig. 6): €7,256,636 (mean €2,419/patient; SD €5,003 – representing 14.3% of total costs), €26,283,138 (mean €8,865/patient; SD €18,435 – representing 22.2% of total costs), and €38,235,840 (mean €12,899/patient; SD €30,151 – representing 24.1% of total costs) at 1, 3, and 5 years, respectively. Overall, the cost savings of anti-TNF therapy per patient were similar when considering 3,000 or 10,000 patients with CD.

**Table 7:** Drug costs of anti-TNF therapy among 3,000 patients with empirical dose escalation compared with a test-based strategy

Cohort of 3,000 patients	1 year	Mean (SD) per patient (1 year)	3 years	Mean (SD) per patient (3 years)	5 years	Mean (SD) per patient (5 years)
Empirical dose escalation	€50,617,472	€16,872/patient (€2,748)	€117,869,125	€39,290/patient (€12,163)	€158,583,140	€52,861/patient (€20,890)
Test-based strategy	€43,360,835	€14,454/patient (€4,180)	€91,630,986	€30,425/patient (€13,583)	€120,347,300	€39,962/patient (€21,741)

### Sensitivity analysis

After a stochastic sensitivity analysis (30 simulations with random choice of transition probabilities and a bootstrap analysis with 3,000 replications of the sample of 30 simulations), these results were comparable in terms of cost savings at 5 years for each patient who was tested for trough levels and antibodies against anti-TNF therapy (95% CI, €13,251.74–13565.05) (Fig. 3).

As stated above, we analyzed costs related to a test-based strategy using an ELISA from Theradiag (at a cost of €100 per test). Several tests have been developed to measure TRI and ATI using ELISA or another method (homogeneous mobility shift assay), with different costs. There was no significant impact of the cost of the diagnostic test on our results until the price reached €2,000 per test (data not shown).

### Cost of the two strategies in 10,000 patients when including costs of postoperative use of anti-TNF therapy and of surgery

We did not take into account either the cost of postoperative use of anti-TNF therapy or the cost of surgery, as the primary aim was to compare the cost of anti-TNF therapy in the case of LOR to IFX. We thus added the cost of surgery (€12,000 per surgical procedure in France) in CD patients with clinical failure of both IFX and ADA according to the treatment algorithm used in our model. It is estimated that about one-quarter of patients will receive anti-TNF therapy after surgery to prevent postoperative recurrence. We chose IFX as postoperative anti-TNF therapy, which was maintained until the end of the 5-year follow-up (27). The number of surgical procedures was higher in the cohort of patients with empirical dose escalation than in those with a test-based strategy (2,011 vs. 1,357 cases, respectively). Surgery was consistently performed earlier in the test-based strategy group than with the empirical optimization strategy (mean duration of anti-TNF therapy: 167±75 weeks vs. 210±58 weeks, respectively).

Cost savings at 5 years, including preoperative and postoperative use of anti-TNF therapy as well as surgery, were €106,437,792 for a cohort of 10,000 patients at 5 years using a test-based strategy.

## Discussion

Recently, Velayos et al. (28) used a decision analytic model (Markov model) that simulated two cohorts of patients with CD and compared outcomes for the following two strategies over a 1-year time period: empirical strategy and test-based strategy. The latter was used to determine simultaneous anti-IFX antibody and residual IFX levels to monitor anti-TNF therapy. A test-based strategy was a cost-effective alternative to the current strategy of empirical dose escalation for managing patients with CD who have lost responsiveness to IFX. The basis for this difference is lower cost for similar outcomes. Velayos et al. (28) reported no data beyond 1 year for IBD, which is known to be a chronic condition requiring long-term immunosuppressive treatment.

Pharmacokinetics is increasingly used to optimize anti-TNF therapy in IBD. However, the cost savings of anti-TNF therapy associated with the use of pharmacokinetics have never been investigated beyond 1 year in a large cohort of CD patients who lose response to IFX therapy. In addition, a decision analytic (Markov) model is not appropriate to address this issue as it is crucial to take into account previous states at any time along the patient's path (28). In our study, a test-based strategy was associated with major cost savings of anti-TNF therapy among CD patients (€131,300,293 for 5 years in a cohort of 10,000 patients). The size of the cohort, 3,000 versus 10,000 patients, did not change our results. Importantly, only the direct costs of anti-TNF therapy in addition to the cost of the test were taken into account in our model. These findings should be taken into account to guide decision making in clinical practice and by health care authorities.

Whether these findings can be extrapolated to other countries will require further investigation. Recently, vedolizumab was approved for refractory IBD, but Sands et al. (24) showed that it was not more effective than placebo in inducing clinical remission at Week 6 in patients who failed anti-TNF therapy. In Europe, certolizumab and natalizumab are not approved for CD, which is why, in case of failure of both ADA and IFX, surgery was performed in our model.

Two studies in IBD patients reported that individualized therapy is more cost-effective than dose intensification in patients with CD who lose response to anti-TNF treatment (28, 29). In a randomized, controlled trial, a therapeutic drug monitoring–based algorithm resulted in improved outcomes and lower cost, compared with empirical management of patients with secondary LOR to IFX (29). The cost effectiveness of a therapeutic drug monitoring (TDM)-based management approach was compared with conventional empirical dose intensification in a randomized, controlled, single-blind multicenter trial (29). Although efficacy was similar for the two strategies (response rates 58 and 53% in the TDM group and standard group, respectively), TDM was more cost effective than empirical dose intensification ($7,736 per patient vs. $11,760 per patient treated, *p*<0.001) due to discontinuation of treatment because of inefficacy in some patients. However, the duration of the study was only 12 weeks (29). Moreover, TRI and ATI were analyzed by radioimmunoassay in the study by Steenholdt et al., whereas we considered an ELISA in our model. However, different assays resulted in similar classification according to the proposed algorithm in the majority of patients (72–78%) and the overall study results were not influenced by the type of analytical technique (30).

A decision analytic model that simulated two cohorts of patients with CD compared outcomes for the two strategies over a 1-year time period (28). In this study, the authors concluded that a test-based strategy is a cost-effective alternative to a strategy of empirical dose escalation in managing patients with CD who have lost responsiveness to IFX. This was a cost-effectiveness study, meaning that they analyzed all costs related to CD treatment in their model (surgery, diagnostic tests, health states, etc.). They also considered certolizumab and natalizumab, which are available in the United States.

Interestingly, by including the costs of postoperative anti-TNF treatment and of surgery, cost savings were still dramatically high in our model: €106,437,792 for a cohort of 10,000 patients at 5 years.

In a recent study, the authors evaluated the cost-effectiveness of personalized treatment of rheumatoid arthritis using clinical response and serum ADA levels (31). Outcomes were simulated using a patient-level Markov model. Clinical effectiveness was higher for the cohort simulated to receive personalized care compared with usual care, and cost savings on drugs were €2,314,354. Testing costs amounted to €10,872.

We found that the cost of the test did not influence our results until it reached €2,000 per test. This finding should be taken into account by health care authorities when discussing the reimbursement of these tests.

Our study has several strengths and some limitations. For the first time, we are reporting the cost savings of anti-TNF therapy in CD patients using a test-based strategy beyond 1 year in large cohorts of patients. Moreover, we used a modeling approach that is appropriate for evaluating such cost savings on drugs. Furthermore, the inclusion of costs of postoperative anti-TNF treatment and of surgical procedures only slightly reduced the cost savings. However, even though all clinical scenarios were considered following a comprehensive literature search, variations in the definition of *clinical response* across studies and differences in treatment algorithms proposed so far based on pharmacokinetic data may have influenced the results yielded by our model.

The major limitation of our study is inherent to the use of modeling techniques, which simplify the real world. Another limitation is that the reliability of the results is only as good as that of the literature data.

In conclusion, a test-based strategy leads to major cost savings related to anti-TNF therapy in CD patients who lose response to IFX: €131,300,293 at 5 years in a cohort of 10,000 patients. As more than 3 million patients have been exposed to anti-TNF agents worldwide for immune-mediated inflammatory diseases and biologics are increasingly used to treat these patients, our findings should be used to reduce health care costs related to anti-TNF therapy. Whether our data can be extrapolated to other health care systems has yet to be determined.

## Key messages

1. A test-based strategy leads to major cost savings related to anti-TNF therapy in Crohn's disease.
2. The direct cost of the test had no impact on the results until the cost per test reached €2,000.
